# Extracting clinical named entity for pituitary adenomas from Chinese electronic medical records

**DOI:** 10.1186/s12911-022-01810-z

**Published:** 2022-03-23

**Authors:** An Fang, Jiahui Hu, Wanqing Zhao, Ming Feng, Ji Fu, Shanshan Feng, Pei Lou, Huiling Ren, Xianlai Chen

**Affiliations:** 1 Life Science College, Central South University, No. 932 South Lushan Road, Changsha, 410083 China; 2 Institute of Medical Information, Chinese Academy of Medical Sciences, No. 3 Yabao Road, Beijing, 100020 China; 3 Dongcheng District, Peking Union Medical College Hospital, No. 1 Shuaifuyuan, Beijing, 100730 China; 4 Big Data Institute, Central South University, No. 932 South Lushan Road, Changsha, 410083 China; 5 National Engineering Lab for Medical Big Data Application Technology, Central South University, No. 932 South Lushan Road, Changsha, 410083 China

**Keywords:** Clinical information extraction, Pituitary adenomas, Chinese electronic medical records, Clinical named entity recognition, Deep learning

## Abstract

**Objective:**

Pituitary adenomas are the most common type of pituitary disorders, which usually occur in young adults and often affect the patient’s physical development, labor capacity and fertility. Clinical free texts noted in electronic medical records (EMRs) of pituitary adenomas patients contain abundant diagnosis and treatment information. However, this information has not been well utilized because of the challenge to extract information from unstructured clinical texts. This study aims to enable machines to intelligently process clinical information, and automatically extract clinical named entity for pituitary adenomas from Chinese EMRs.

**Methods:**

The clinical corpus used in this study was from one pituitary adenomas neurosurgery treatment center of a 3A hospital in China. Four types of fine-grained texts of clinical records were selected, which included notes from present illness, past medical history, case characteristics and family history of 500 pituitary adenoma inpatients. The dictionary-based matching, conditional random fields (CRF), bidirectional long short-term memory with CRF (BiLSTM-CRF), and bidirectional encoder representations from transformers with BiLSTM-CRF (BERT-BiLSTM-CRF) were used to extract clinical entities from a Chinese EMRs corpus. A comprehensive dictionary was constructed based on open source vocabularies and a domain dictionary for pituitary adenomas to conduct the dictionary-based matching method. We selected features such as part of speech, radical, document type, and the position of characters to train the CRF-based model. Random character embeddings and the character embeddings pretrained by BERT were used respectively as the input features for the BiLSTM-CRF model and the BERT-BiLSTM-CRF model. Both strict metric and relaxed metric were used to evaluate the performance of these methods.

**Results:**

Experimental results demonstrated that the deep learning and other machine learning methods were able to automatically extract clinical named entities, including symptoms, body regions, diseases, family histories, surgeries, medications, and disease courses of pituitary adenomas from Chinese EMRs. With regard to overall performance, BERT-BiLSTM-CRF has the highest strict F1 value of 91.27% and the highest relaxed F1 value of 95.57% respectively. Additional evaluations showed that BERT-BiLSTM-CRF performed best in almost all entity recognition except surgery and disease course. BiLSTM-CRF performed best in disease course entity recognition, and performed as well as the CRF model for part of speech, radical and document type features, with both strict and relaxed F1 value reaching 96.48%. The CRF model with part of speech, radical and document type features performed best in surgery entity recognition with relaxed F1 value of 95.29%.

**Conclusions:**

In this study, we conducted four entity recognition methods for pituitary adenomas based on Chinese EMRs. It demonstrates that the deep learning methods can effectively extract various types of clinical entities with satisfying performance. This study contributed to the clinical named entity extraction from Chinese neurosurgical EMRs. The findings could also assist in information extraction in other Chinese medical texts.

## Background

Pituitary adenomas are one of the most common intracranial tumors, accounting for about 10% of intracranial tumors [1, 2]. The population incidence rate is 1/100,000–7/100,000 [3, 4]. In recent years, the incidence rate has increased significantly, and the onset age is younger. In China, with a population of 1.3 billion, there are 13,000–91,000 new cases each year, and the actual prevalence is much higher than that. The detection rate of pituitary adenomas in autopsy is as high as 20–30% [4]. This type of tumor occurs in the intracranial human endocrine center, i.e., the pituitary gland. It would severely damage the physical and mental health of patients, such as physical development, labor capacity, fertility and so on. Unfortunately, the pathogenesis of pituitary adenomas has not been fully understood so far.

The extensive and practical clinical information contained in the electronic medical records (EMRs) of pituitary adenomas can be used for early detection of the disease. There are various types of clinical texts. Generally, a patient’s text medical records include admission notes, course notes, surgical notes, discharge notes, etc. These clinical notes have great potential for in-depth exploration and medical knowledge reuse value. Existing studies have shown that the analysis and mining of EMRs texts can promote applications such as disease prediction, drug discovery, auxiliary diagnosis and treatment, and medical record retrieval [5–8]. The in-depth mining of EMRs of pituitary adenomas will helpful for further clinical study of pituitary tumors, and the clinical information contained in the EMRs of pituitary adenomas will beneficial for promoting potential medical discoveries.

The free-text form of information expression provides convenience for medical staff to write medical records, but brings great challenges to automatic analysis and acquisition of clinical information. Extracting meaningful clinical information from free texts is the most critical and fundamental process for medical data analysis and mining.

In this paper, we study the methods of clinical information extraction for pituitary adenoma. Several information extraction methods are compared and contrasted, including dictionary-based matching, conditional random fields (CRF), bidirectional long short-term memory with CRF (BiLSTM-CRF) and bidirectional encoder representations from transformers with BiLSTM-CRF (BERT-BiLSTM-CRF). Our goal is to train and find the best clinical information extraction model for pituitary adenoma based on Chinese EMRs by evaluating the above mentioned methods. To the best of our knowledge, this is the first study on the clinical information extraction method of pituitary adenoma based on Chinese EMRs.

## Related works

Information extraction has been receiving a lot of research attention, since freely expressed text data contains rich useful information. Previous studies have validated the feasibility of identifying and extracting information from texts in clinical fields. Most of the early information extraction used rule-based or dictionary-based methods. In recent years, with the development of machine learning, more and more studies use neural networks for clinical information extraction. Mykowiecka et al. [9] utilize a rule-based method to extract information from patients’ clinical data. Obeid et al. [10] detected mental status in emergency department clinical notes adopting the convolutional neural network (CNN). Su et al. [11] extracted risk factors for cardiovascular diseases by training BLSTM-CRF-based model. Zhang et al. [12] used fine-tuned BERT to extract clinical information from breast cancer-based clinical texts.

The information processing methods in the open evaluation tasks can be used as references for clinical information processing. Comparing with the open data, EMRs data has stronger privacy requirements [13]. With the development of EMRs related open evaluation tasks, more and more researchers are involved in clinical information extraction tasks, which lead to a wider range research of clinical natural language processing (CNLP). Therefore, analyzing the methods used in the frontier CNLP evaluation tasks is beneficial for the study of clinical information extraction for pituitary adenomas. The clinical information extraction evaluation based on English EMRs is represented by Informatics for Integrating Biology & the Bedside/National NLP Clinical Challenges (i2b2/n2c2) and Shared Annotated Resources/Conference and Labs of the Evaluation Forum (SAR/CLEF) eHealth Evaluation Lab. The evaluation tasks of the clinical information extraction over the years include different types of entity recognition, clinical information classification, clinical terminology standardization, and automatic screening of clinical trials [14–16].

Compared with English clinical information processing, the research of Chinese clinical information extraction started relatively late. Recently, clinical entity recognition based on Chinese EMRs has received extensive attention [17–19], and related evaluation tasks have been carried out continuously. China Conference on Knowledge Graph and Semantic Computing (CCKS) is one of the most representative organizations providing various open evaluations based on Chinese clinical texts. CCKS has initiated the task of Chinese clinical named entity recognition since 2017. The clinical entities of the CCKS 2017 evaluation task include symptoms and signs, examinations and tests, diseases and diagnosis, treatment, and body regions. The CCKS 2018 evaluation task focuses on anatomical parts, description of symptoms, independent symptoms, medication, and surgery. Based on the evaluation tasks of the previous two years, CCKS 2019 was interested in disease and diagnosis, examination, inspection, surgery, medication, and anatomy. From the related studies of clinical information extraction, it can be found that admission notes, disease course notes, and discharge summaries are the main objects of clinical information extraction [20, 21], which contain a large number of clinical entities.

With the advancement and development of technology, various methods of information extraction systems have been developed. The traditional methods are Hidden Markov Model (HMM), CRF and Support Vector Machine (SVM), among which the CRF model has achieved remarkable results in the field of information extraction [22]. With the development of neural networks and deep learning, more and more information extraction studies [23, 24] combined deep learning with traditional rules and other machine learning methods, such as long short-term memory with CRF (LSTM-CRF) and BiLSTM-CRF. The Bidirectional Encoder Representations from Transformers (BERT) [25] was proposed by the Google AI team in November 2018, which has achieved exciting performances in natural language processing related tasks. In the information extraction and evaluation tasks of CCKS 2019, almost all evaluation teams integrated this model and achieved remarkable results.

## Materials and methods

### Dataset

#### Clinical notes

**Fig. 1:**
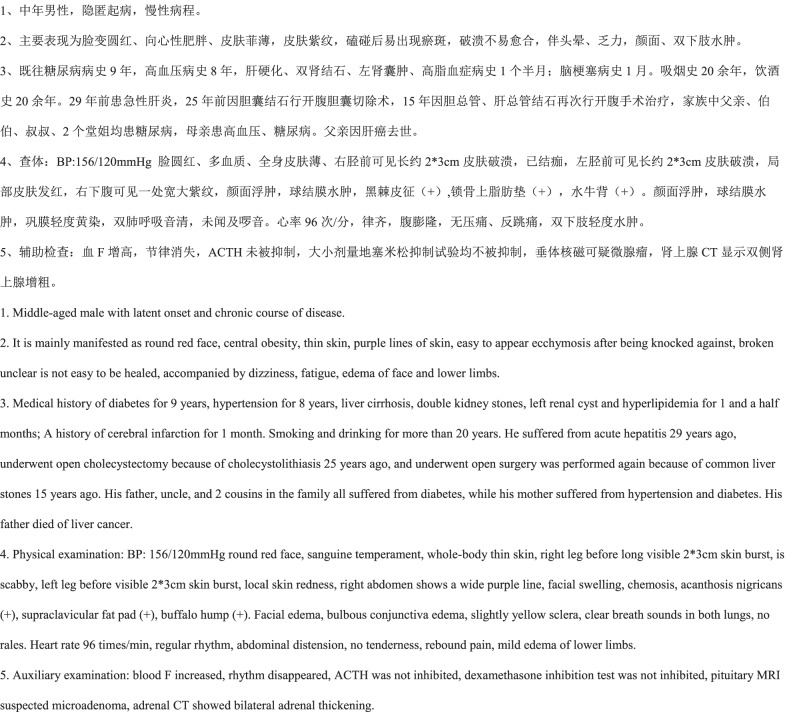
An example of the original EMR texts of pituitary adenomas

For each patient, EMRs contain a series of clinical notes, among which the admission notes, the first-time progress notes and the discharge notes are focused as the three key objects for the research of clinical information extraction. Admission notes completed within 24 h of admission are generally used to record the patients’ complaints and symptoms, medical history, marriage status, family history and basic vital signs, etc. The first-time progress notes contain the basic conditions of the patients within 8 h of the admission, including clinical case characteristics, diagnosis discussion, differential diagnosis, and treatment plan. Discharge notes summarize the patients’ treatment, including hospitalization, admission diagnosis, treatment plan, discharge diagnosis, discharge status and discharge instructions. There is information redundancy between different notes, e.g., hospitalization condition in discharge records are usually the same as chief complains and past history in the admission notes. Through the comprehensive analysis of these different clinical notes, we selected four fine-grained texts types of clinical records, including the current medical histories, past medical histories, case characteristics and family histories of 500 pituitary adenoma inpatients. Figure 1 shows an example of the original EMR texts of pituitary adenomas.

#### Entities of interest

Seven entity types including symptoms, body regions, diseases, family histories, surgeries, medications, and disease courses were determined through in-depth discussions with experienced neurosurgeons for extracting the clinical information of pituitary adenomas. Table 1 shows an example of the clinical information extracted from the example of original EMR texts of pituitary adenomas presented in Fig. 1. The entities in Table 1 correspond to the annotations of the text in Fig. 1.

- Symptoms are of vital priority in the diagnosis, referring the patients’ subjective feeling of discomforts and the pathological reaction of objective examinations. Negative symptoms (e.g., no nausea, vomiting, deny of fever), typical disease symptoms (e.g., nose hypertrophy, moon face), and relieved symptoms after medication or surgery (e.g., tinnitus slightly reduced, spirit better than before) should be extracted.
- Body regions refer to the anatomic sites involved with patients’ complains and symptoms, such as abdomen, neck, and temporal side.
- Diseases refer to the complicated processes including quantitative and qualitative change in many cases. In the study, diseases refer to the abnormal examination findings and diagnosis mentioned in the medical records, including routine examination diseases (e.g., hypertension, hyperlipidemia), diagnosis-related diseases (e.g., pituitary tumor, deafness, dry eye) and abnormal examination findings (e.g., saddle area occupation, sphenoiditis). For the patient’s disease, the medical history of the patient's own related diseases should be extracted as disease. Diseases and medical histories mentioned in the text that are not the patient's own (which are other members of the family) should be taken as family history.
- Family histories refer to the genetic diseases that other family members suffer from. Only the positive diseases will be extracted, such as diabetes, lung cancer, heart diseases, etc., which have a certain heredity generally. Similarly, negative statement of diseases such as “no hypertension” and “no diabetes” stated in the medical records were not included in the scope of family histories.
- Surgeries refer to the patients’ operation histories recoded in the medical records, such as transsphenoidal sinus surgery, pituitary tumor resection operation, etc.
- Medications refer to medications prescribed to patients recorded in the medical records, such as glucocorticoids, reserpine, aspirin, diazepam, euthyrox, etc.
- Disease courses record the development status or duration of the patient's disease, such as chronic course, insidious onset, two-year course, etc., which have significant clinical research value.

**Table 1:** Examples of the clinical named entities extracted from EMR texts of pituitary adenomas

Entities	Start positions	End positions	Entity types
隐匿起病 (latent onset)	7	11	Disease course
慢性病程 (chronic course of disease)	12	16	Disease course
向心性肥胖 (centripetal obesity)	30	35	Symptom
皮肤菲薄 (thin skin)	36	40	Symptom
双下肢 (lower limbs)	72	75	Body region
水肿 (edema)	75	77	Symptom
糖尿病病史 (medical history of diabetes)	83	88	Disease
高血压病史 (medical history of hypertension)	91	96	Disease
肝硬化 (liver cirrhosis)	99	102	Disease
双肾结石 (double kidney stones)	103	107	Disease
左肾囊肿 (left renal cyst)	108	112	Disease
开腹胆囊切除术 (open cholecystectomy)	168	175	Surgery
皮肤发红 (skin redness)	314	318	Symptom
锁骨上脂肪垫 (supraclavicular fat pad)	350	356	Symptom
水牛背 (buffalo hump)	360	363	Symptom
巩膜 (sclera)	378	380	Body region
黄染 (yellow)	382	384	Symptom
腹膨隆 (abdominal distension)	409	412	Symptom
微腺瘤 (microadenoma)	480	483	Disease
双侧肾上腺增粗 (bilateral adrenal thickening)	491	498	Disease

#### Annotated corpus

Two assistants majoring in neurosurgery were trained to annotate the dataset. The inter-annotator agreement (IAA) is calculated using the F1 value. The annotation result of one annotator (A1) is regarded as the standard answer, and the precision (P) and recall rate (R) of the annotation result of another annotator (A2) are calculated. Then we can calculate the F1 value, and the calculation formula is as following:

$$P = \frac{The\, total\, number\, of\, annotation\, results\, that\, are\, consistent\, with \,A1 \,and \,A2}{{The\, total\, number\, of\, annotations\, for\, A2}}$$

$$R = \frac{The\, total\, number\, of\, annotation\, results\, that\, are\, consistent\, with\, A1 \,and\, A2}{{The\, total\, number\, of\, annotations\, for\, A1}}$$

$$F1 = \frac{2*P*R}{{P + R}}$$

The IAA value of the entity annotation was 0.896. According to the quality requirements of reliable corpus [26], when the consistency reaches 0.8, the consistency of the corpus can be considered valid and reliable. So our clinical named entity corpus for pituitary adenomas is reliable in consistency and meets the quality requirements. To provide a higher quality corpus for information extraction in this study, we added a senior pituitary tumor expert as the reviewer to unify the inconsistent annotation results on the basis that the IAA value annotated by multiple annotators meets the requirements.

As mentioned above, the dataset contains four types of clinical notes, including the current medical histories, past medical histories, case characteristics and family histories of 500 pituitary adenoma inpatients. For the experimental study, annotated records of 300 patients were selected as training set, 100 records were used as test set, and the remaining 100 records were used for validation in the training process of the two BiLSTM-CRF models. The token distribution of the seven types of entities included in the training, validation and test set are shown in Table 2. The token distribution of the seven types of entities included in the current medical history, past medical history, case characteristics and family history are shown in Table 3.

**Table 2:** Token distribution of the seven types of entities in three kinds of dataset

Entity	Training set	Validating set	Testing set
Symptom	10,880	3633	3655
Body region	981	451	507
Disease	3760	1260	1339
Surgery	616	215	165
Medication	742	205	197
Family history	137	46	61
Disease course	281	82	104
All	17,367	5892	6028

**Table 3:** Token distribution of the seven types of entities in four kinds of EMR texts

Entity	Current medical history	Past medical history	Case characteristics	Family history
Symptom	14,006	180	3982	0
Body region	929	39	971	0
Disease	1609	3733	984	3
Surgery	123	605	268	0
Medication	673	217	154	0
Family history	1	2	45	196
Disease course	0	0	467	0
All	17,341	4876	6871	199

### Methods

Clinical named entity recognition can be transformed into a sequence labeling task aiming at identifying and classifying entities in the clinical texts. The annotated corpus is in JSON format, and it needs to be converted into BIO format to represent the boundary of entities. Unlike English or other western languages separated by space, Chinese characters do not have clear boundaries and capitalization. Chinese word segmentations may bring some errors, which further affect the accuracy of named entity recognition. Recent studies [27, 28] indicated that character-based named entity recognition methods in Chinese corpus are much more effective than the word-based methods. We use BIO format to assign each character a label, where O represents non-medical entity category, B represents the beginning of the entity, and I represents the middle or end of the entity. There were 7 entity categories in this study, namely symptoms, body regions, diseases, family histories, surgeries, medications, and disease courses, corresponding to 15 labels totally, i.e., B-symptom (Symptom in Table1), B-body (Body region in Table1), B-disease (Disease in Table1), B-family (Family history in Table1), B-surgery (Surgery in Table1), B-medication (Medication type), B-progress (Disease course in Table1), I-symptom, I-body, I-disease, I-family, I-surgery, I-medication, I-progress and O.

**Fig. 2:**
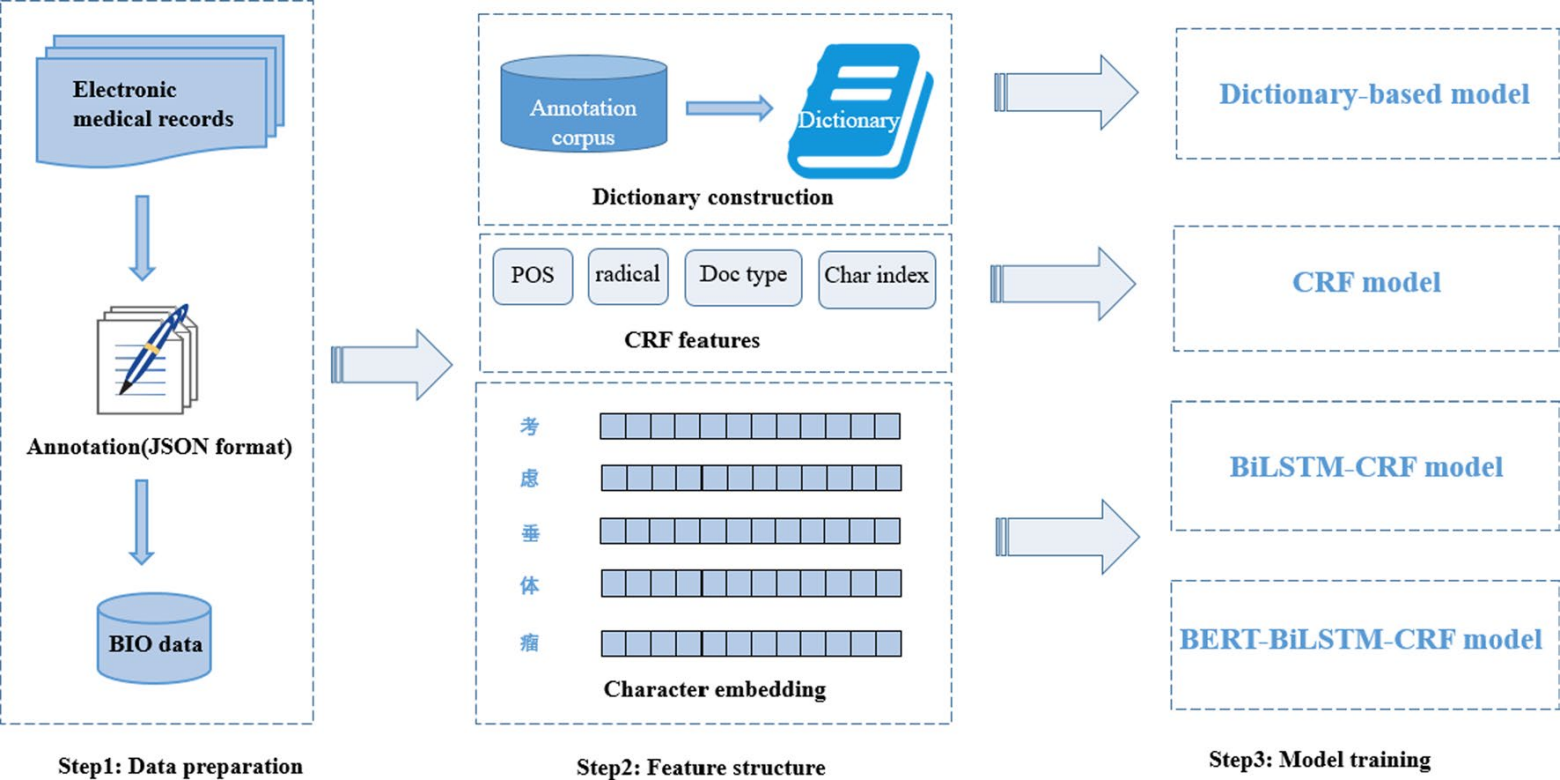
The processing flow of our methods

Four medical named entity recognition methods were adopted in this paper, including dictionary-based matching model, CRF model, BiLSTM-CRF model and pretrained BERT character embeddings-bidirectional long short-term memory with CRF (BERT-BiLSTM-CRF) model. Figure 2 shows the processing flow of our methods. After the Chinese EMRs were annotated into JSON format and converted into BIO format, we constructed a dictionary by using part of the annotated corpus, selected POS, radical, document type (Doc type) and the position of characters (Char index) as CRF features, and character embeddings as input features for the BiLSTM-CRF model and the BERT-BiLSTM-CRF model. In the Fig. 2, the example sentence “考虑垂体瘤” means “consider pituitary tumors”.

#### Dictionary-based methods

1. Comprehensive dictionary based on open source vocabulary

The rationale for the dictionary-based method is to maximize string matching. Our initial consideration was to construct a large dictionary of medical entities. The open sources chosen in this study include: the open source Chinese symptom database knowledge graph,^1^ the series of challenge evaluations of CCKS and CHIP, and pituitary adenomas-related diseases and symptoms appeared in Chinese Baidu encyclopedia. Unfortunately, the accuracy rate obtained by this method was very low (under 20%). After error analysis, it was found that many errors might be introduced since the selected dictionary sources were not specific to pituitary adenomas, but in a wider range of general medical fields, many entities extracted by the dictionary-based method were not within the scope of interest in this study, e.g., “肿 (swell)”, “精神病 (mental disease)”. In addition, there were also some lack-of-words errors, e.g., “慢性病 (chronic disease)”, “瘤病 (tumor disease)”.

(2) Neurosurgery domain dictionary

**Fig. 3:**
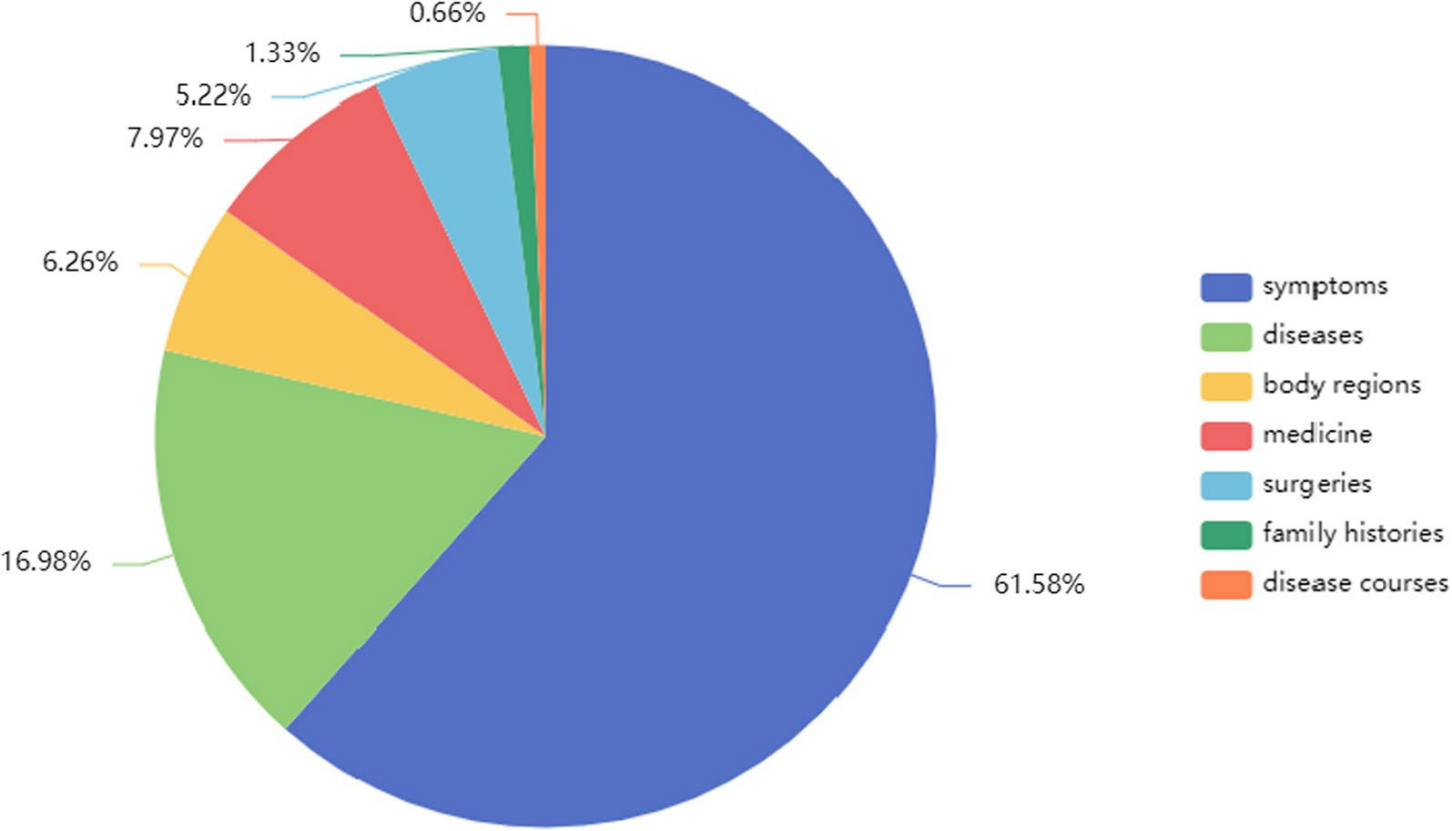
Entities distribution in the domain dictionary

The annotated notes of 50 pituitary adenoma inpatients are used to construct a domain dictionary. This dictionary contains a total of 1,054 entities, including 649 symptoms, 66 body regions, 179 diseases, 84 surgeries, 55 medications, 14 family histories, and 7 disease courses, as shown in Fig. 3. The dictionary-based named entity recognition in this paper is based on this domain dictionary using maximum string matching.

#### CRF model

CRF is a probabilistic structure model used for labeling and dividing sequence structure data. In this study, model defines the conditional probability $P(Y|X)$ according to the random variable over clinical data sequences $X$ and random variable over corresponding marking label sequences $Y$.

Let $G = (V,E)$ is an undirected graph, consisting of the set of nodes $V$ and undirected edges $E$. For a given data sequence $X$, let the corresponding label sequences $Y = \{ Y_{v} |v \in V\}$, where $Y_{v}$ (a random variable of each node $V$) satisfies Markov characteristics $p(Y_{v} |X,Y_{w} ,w \ne v) = p(Y_{v} |X,Y_{w} ,w\sim v)$, where $w\sim v$ represents the neighboring node in graph $G$, then $(X,Y)$ is a conditional random field.

For a given data sequence $X$, the probability of the corresponding label sequences $Y$ is $P(Y|X) = \frac{1}{Z(x)}\exp (\sum\limits_{j} {\lambda_{j} f_{j} (y_{i - 1} ,y_{i} ,x)} + \sum\limits_{k} {\mu_{k} g_{k} (y_{i} ,x)} )$, where $Z(x)$ is the normalization factor, and $\lambda_{j}$,$\mu_{k}$ are the weights for each feature function, $f_{j}$ is the feature function of current label $y_{i}$ and previous label $y_{i - 1}$, $g_{k}$ is the feature function of current label $y_{i}$. The parameter training process can be performed on the training data set based on the maximization of the log-likelihood function.

#### BiLSTM-CRF model

Text needs to be convert into vector form to use deep learning models. A simple sentence like “考虑垂体瘤,…,无头晕 (consider pituitary tumors, …, no dizzy)” can be represented as a one-hot matrix, where each character is an one-hot vector, and then transformed into character embedding. The character embedding matrix is initialized randomly. Since Word2vec embedding does not consider the word orders, poor entity recognition performance is got in our dataset.

The LSTM layer can efficiently use long-distance information. At $t$ time, given input $x_{t}$, the specific calculation process represented by output of hidden layer of LSTM is as follows:

$$i_{t} = \sigma ({\mathbf{W}}_{xi} x_{t} + {\mathbf{W}}_{hi} h_{t - 1} + {\mathbf{W}}_{ci} C_{t - 1} + {\mathbf{b}}_{i} )$$

$$f_{t} = \sigma ({\mathbf{W}}_{xf} x_{t} + {\mathbf{W}}_{hf} h_{t - 1} + {\mathbf{W}}_{cf} C_{t - 1} + {\mathbf{b}}_{f} )$$

$$C_{t} = f_{t} C_{t - 1} + i_{t} tanh({\mathbf{W}}_{xC} x_{t} + {\mathbf{W}}_{hC} h_{t - 1} + {\mathbf{b}}_{C} )$$

$$o_{t} = \sigma ({\mathbf{W}}_{xo} x_{t} + {\mathbf{W}}_{ho} h_{t - 1} + {\mathbf{W}}_{co} C_{t} + {\mathbf{b}}_{o} )$$

$$h_{t} = o_{t} tanh(C_{t} )$$

where ${\mathbf{W}}$ represents the weight matrix (${\mathbf{W}}_{xi}$ represents the weight matrix of the input gate from the input layer to the hidden layer), ${\mathbf{b}}$ is the offset vector (${\mathbf{b}}_{i}$ represents the bias vector of the input gate of the hidden layer), $C$ is the state of a memory unit, $\sigma$ and $tanh$ are two different neuronal activation functions, $i_{t}$, $f_{t}$ and $o_{t}$ represents input gate, forgetting gate and output gate respectively, $h_{t}$ is the context vector of a character. The threshold mechanism can effectively filter and memorize the information of memory unit. We use bidirectional LSTM layer to get both past and future input information and extract features automatically.

**Fig. 4:**
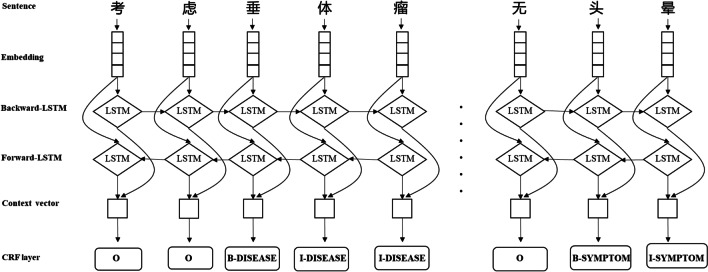
The architecture of BiLSTM-CRF model

In the prediction stage, the softmax layer is usually used to solve the multi-classification problem, but it does not consider the dependency relationship between the labels in the sequence labeling problem, which might get ungrammatical label sequences. Therefore, we use the CRF layer instead. In the output of the CRF model, label I-X does not appear after label O, because label I-X must be connected after label B-X. This error may occur in the softmax layer, where the label of the first text may be predicted to be O and the label of the latter to be I-X. Thus, we inputted the features learned by BiLSTM into the CRF layer to avoid grammar problems and to improve the accuracy of recognition due to the diversity of features. The architecture of BiLSTM-CRF model is shown in Fig. 4.

#### BERT-BiLSTM-CRF model

**Fig. 5:**
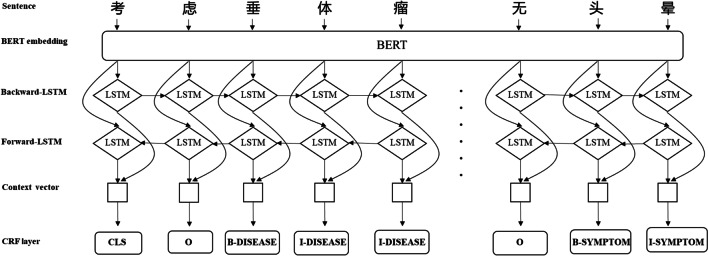
The architecture of BERT-BiLSTM-CRF model

BERT-BiLSTM-CRF model is similar as BiLSTM-CRF model, the only difference is the input word vector. A BERT model is added to the BiLSTM model as the feature presentation layer and used to generate word vectors, which are the input of LSTM structure. The architecture of BERT-BiLSTM-CRF model is shown in Fig. 5.

## Experiments

### Experimental setup

The CRF++ tool is used to train the CRF model, and four features (i.e., POS, radical, document type and character index) are selected, then different feature templates were written based on multi-feature fusion. The POS of the character is defined as the POS of the word which the character belongs to. The character-level POS tags are generated by jieba segment system. The radical tags are generated by xmnlp library, such as “讠”, “疒”, labeled as “N” for character without radical like Latin alphabet, numbers, and punctuations, etc. There are four types of clinical text document, i.e., the current medical history, past medical history, case characteristics and family history, which are denoted by “current”, “past”, “family” and “case” respectively; character index indicates the position of the character in this paragraph, counting from 0. The content window size is set as 5, which means the two positions before and after the current position are used as the marks to constitute the feature model, and then 16 unigram templates are designed for entity extraction.

For BiLSTM-CRF model, character embedding size is set as 100, hidden dimension as 100, maximum training epoch as 30, batch size as 16, learning rate as 0.01, clip as 5, and dropout rate as 0.5. The optimizer is set to Adam.

For BERT-BiLSTM-CRF model, BERT-Base pre-trained language model is uesd with 12 layers, 768 hidden layer dimension, 12-head mode and 110M parameters. Maximum sequence length is set as 128, training epoch as 40, batch size as 32, learning rate as 1e-5, clip as 0.5, and dropout rate as 0.5.

### Evaluation criteria

We use CCKS evaluation standard as criteria, the correct entities labeled in the strict evaluation metric are exactly the same as the ground truth, while in the relaxed evaluation metric the entity boundaries are include in the ground truth. We define $S=\left\{{s}_{1},{s}_{2}\dots {s}_{m}\right\}$ as the models output results and $G=\{{g}_{1},{g}_{2}\dots {g}_{n}\}$ as the golden standard. Then ${s}_{i}\in S$ and ${g}_{j}\in G$ are strictly equal only when:

$${s}_{i}.content={g}_{j}.content$$

$${s}_{i}.start={g}_{j}.start$$

$${s}_{i}.end={g}_{j}.end$$

$${s}_{i}.entity\_type={g}_{j}.entity\_type$$

and leniently equal when:

$${s}_{i}.content={g}_{j}.content$$

$$\mathrm{max}\left({s}_{i}.start,{g}_{j}.start\right)\le \mathrm{min}\left({s}_{i}.end,{g}_{j}.end\right)$$^2^

$${s}_{i}.entity\_type={g}_{j}.entity\_type$$

Here, $content$ means the semantic concept, $start$ and $end$ mean the first and last character’s position in the text. Precision (P), Recall (R) and F1 score are calculated by the following formulas:

$$P = \frac{{\left| {S \cap G} \right|}}{\left| S \right|},\quad R = \frac{{\left| {S \cap G} \right|}}{\left| G \right|},\quad F_{1} = \frac{2PR}{{P + R}}$$

## Results

The text of case characteristics is noted in the first course record, which records the basic information of the patient within 8 h on the day of admission. In addition to the case characteristics, it also records the discussion of possible diagnosis, differential diagnosis and treatment plan. The text of the current medical history is noted in the admission record, which is usually completed within 24 h to record the patient's chief complaint, medical history of present illness, past history, marital and child history, family history, basic physical examination and other information. Our dataset covers medical history of present illness, past history, case characteristics and family history, and each patient has these four types of clinical text.

In the CRF model experiment, four features were selected, namely POS, radical, document type, and the position of characters in addition to the basic CRF model. In order to validate these features, a total of 16 sub-experiments were carried out on the characteristics of types (including basic CRF model). Experimental results of dictionary-based model, 16 CRF models, BiLSTM-CRF model and BERT-BiLSTM-CRF model were shown in Table 4, and we used “+” as the connect symbol of the features in CRF models. BERT-BiLSTM-CRF model performed best in both strict and relaxed overall F1 value and the dictionary-based method had the worst performance. The best strict F1 value was 91.27%. There were two models reaches the second-best strict F1 value, i.e., CRF + pos + radical + type and CRF + pos + type, all of which were 90.42%, superior to the strict F1 value of BiLSTM-CRF model of 89.94%. While comparing the relaxed F1 value, it was found that the BERT-BiLSTM-CRF model had the highest relaxed F1 value of 95.57%, while the BiLSTM-CRF model was 94.67% and CRF + pos + radical + type was 94.41%. The strict F1 value of the CRF basic model reached 89.85%. For CRF features-models, the combination of multiple features, such as POS, radical and document type, can slightly improve the performance, while the word index feature is an exception, which seemed to cause the overall F1 value decreasing.

**Table 4:** Performance comparison of all models

Model	Overall					
	Strict			Relaxed		
	P (%)	R (%)	F1 (%)	P (%)	R (%)	F1 (%)
Dictionary	42.74	43.44	43.08	64.08	65.13	64.60
CRF	92.18	87.63	89.85	96.18	91.43	93.75
+ pos	91.89	88.28	90.05	96.01	92.23	94.08
+ radical	91.90	87.84	89.82	96.00	91.76	93.83
+ type	92.31	87.82	90.01	96.52	91.82	94.11
+ index	91.07	86.16	88.55	95.66	90.51	93.01
+ pos + radical	91.90	87.95	89.88	96.13	92.01	94.03
+ pos + type	92.32	88.60	90.42	96.39	92.50	94.40
+ pos + index	91.08	87.43	89.22	95.62	91.79	93.66
+ radical + type	91.98	87.99	89.94	96.22	92.04	94.09
+ radical + index	91.12	87.18	89.10	95.59	91.45	93.47
+ type + index	91.33	86.37	88.78	96.02	90.79	93.33
+ pos + radical + type	92.43	88.49	90.42	96.51	92.40	94.41
+ pos + radical + index	91.21	87.45	89.29	95.63	91.69	93.62
+ pos + type + index	91.27	87.58	89.39	95.86	91.99	93.89
+ radical + type + index	91.22	87.19	89.16	95.79	91.57	93.63
+ pos + radical + type + index	91.46	87.75	89.57	95.86	91.97	93.88
BiLSTM-CRF	90.24	89.64	89.94	94.98	94.36	94.67
BERT-BiLSTM-CRF	92.00	90.54	**91.27**	96.34	94.81	**95.57**

Bold means best performance of all models

**Fig. 6:**
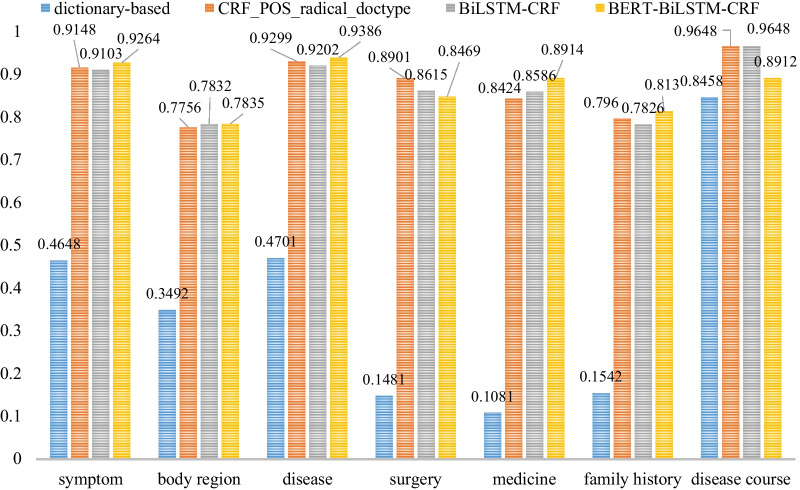
The strict F1 values of the models for seven medical entity types

**Fig. 7:**
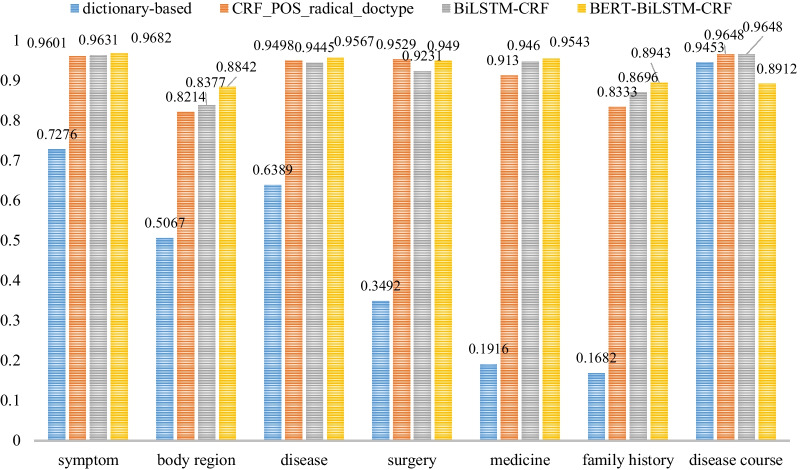
The relaxed F1 values of the models for seven medical entity types

The F1 value represents the performance of each entity extraction model. We further tried to find out the best performed model for each type of entity. Figures 6 and 7 show the detailed performance of each entity type between dictionary-based method, CRF + pos + radical + typeP, BiLSTM-CRF model and BERT-BiLSTM-CRF model both in strict and relaxed F1 value.

The dictionary-based method performed the worst even for each entity type, only the disease course entity recognition was not too bad, arriving at 84.58% with strict F1 value and 94.53% with relaxed F1 value. BERT-BiLSTM-CRF model performed best in almost all entity recognition except surgery and disease course with both strict and relaxed F1 value. BiLSTM-CRF model performed best in disease course entity recognition, which was the same as the CRF + pos + radical + type model, arriving at 96.48% with both strict and relaxed F1 value. The CRF + pos + radical + type model performed best in surgery entity recognition with relaxed F1 value, arriving at 95.29%.

## Discussion

The results show that deep learning and other machine learning methods were able to automatically extract clinical named entity for pituitary adenomas from Chinese EMRs, and BERT-BiLSTM-CRF model performs best in the overall entity recognition but not in every entity. Because the dictionary-based method is based on the maximum string matching, it cannot utilize the context information, so the performance is poor. To discuss the effects and errors for different entity recognition, we focused on CRF features models, BiLSTM-CRF model and BERT-BiLSTM-CRF model studied in this paper.

**Fig. 8:**
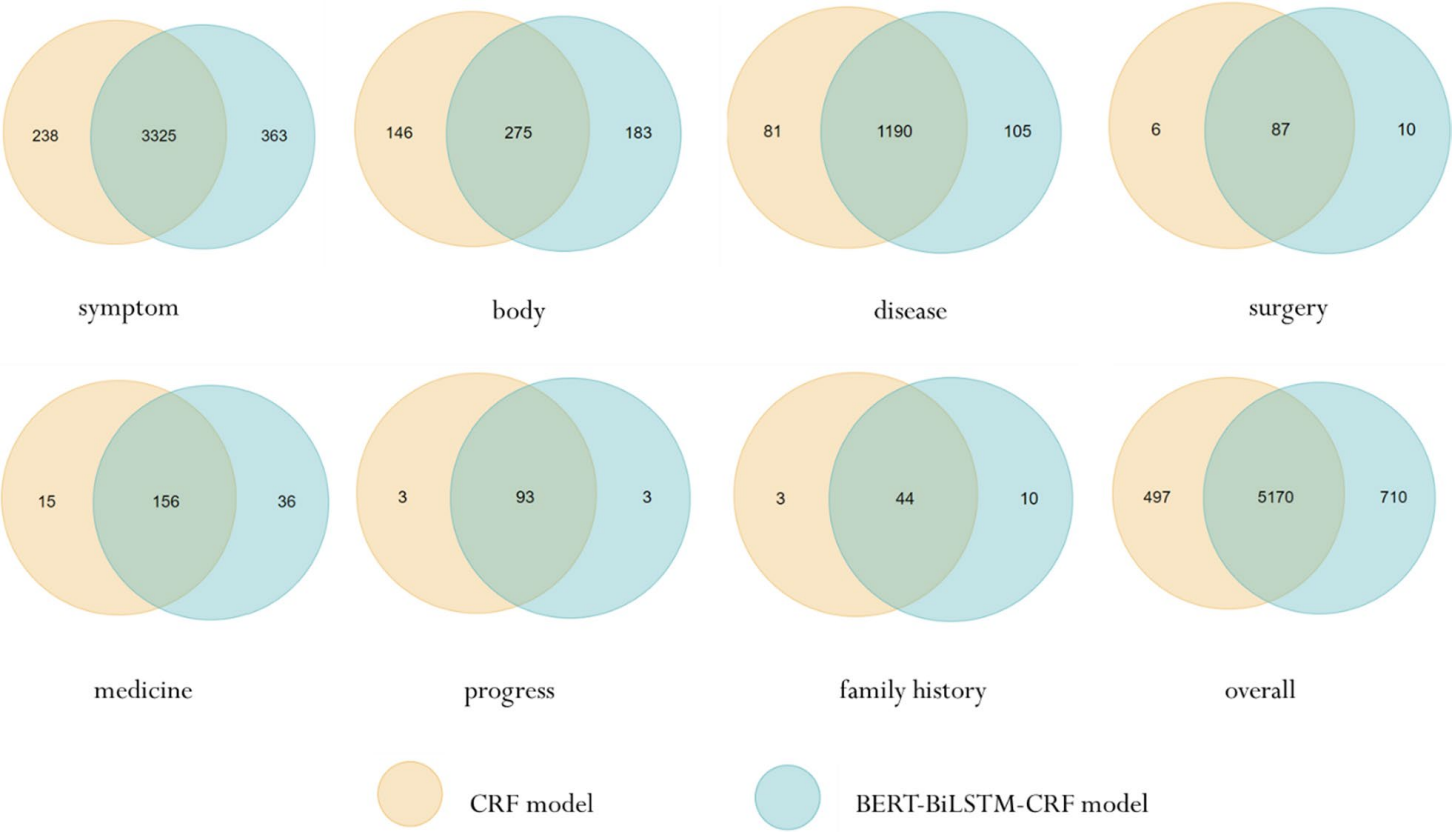
The overlap of tokens between CRF model and BERT-BiLSTM-CRF model

Although our dataset meets the quality requirements of reliable corpus, which is further demonstrated from the experimental results of entity recognition, and the machine learning models have reached high overall F1 values (see Table 4), there still exist some entity recognition errors caused by the inconsistency of annotation. The F1 value of the output results of the CRF model and the BERT-BiLSTM-CRF model is 88.33%, indicating that the two models can find many similar entities, and the overlap of tokens between these two models are shown in Fig. 8. For example, “双下肢水肿 (edema of double lower extremities)” is sometimes labeled as a single entity “symptoms”, while it is sometimes labeled as two entities, namely “双下肢 (double lower extremities)” is labeled as “body region”, and “水肿 (edema)” is labeled as “symptom”. This puts forward certain requirements on the generalization ability of the model. Comparing the results of multiple models, it is found that all models are more inclined to label “双下肢水肿 (edema of double lower extremities)” as “symptom”, while for “双下肢凹陷性水肿 (pitting edema of double lower extremities)”, almost all models can label “双下肢 (double lower extremities)” as “body region” and “凹陷性水肿 (pitting edema)” as “symptom”. Although we tended to annotate more specifically, it is demonstrated that models maybe prefer recognizing long symptom entities than single-character and double-character symptom entities. Similar errors also appear in “手指关节胀痛 (finger joint pains)” and “头顶部跳痛 (top of head throbbing pains)”.

In order to construct a high-quality corpus, the body region entities in our experiment dataset are fine-grained annotated. For example, “双侧视野缺损 (bilateral visual field defect)” is annotated as two entities, “双侧 (bilateral)” is labeled as “body region”, and “视野缺损 (visual field defect)” is labeled as “symptom”. Owing to this approach, the recognition effects of these models are not much different. But the fine-grained annotation is not suitable for all situations, which inevitably bring some problems. For example, when extracting entities from the text “双侧大腿内侧皮肤紫纹 (bilateral inner thigh skin purple streaks)”, “双侧大腿内侧 (bilateral inner thigh)” is expected to be recognized as an independent entity “body region”, while almost all models incorrectly identify it as two or three entities, such as “双侧 (bilateral)”, “大腿内侧 (inner thigh)”, or “大腿 (thigh)” and “内侧 (inner)”.

The CRF feature models do not distinguish well between entities separated by punctuation marks “?”. For example, when extracting entity from “考虑垂体腺瘤伴囊变?蝶窦内炎症? (Considering pituitary adenoma with cystic degeneration? Inflammation in the sphenoid sinus?)”, “垂体腺瘤伴囊变?蝶窦内炎症 (pituitary adenoma with cystic degeneration? Inflammation in the sphenoid sinus)” is recognized as “disease”. This type of error requires subsequent splitting of the entity through certain post-processing operations. Besides, the CRF basic model also tends to extract longer entities in the recognition of drug entities, such as “降压药物富马酸比索洛尔 (hypertensive drug bisoprolol fumarate)”, “地米沙坦+阿司匹林 (dimisartan + aspirin)”. The introduction of POS and radical features reduces such errors.

In addition, it should be noticed that the introduction of document type feature in CRF has greatly improved the recognition of family history entities. This is mainly due to the fact that family history entities mostly appear in “family history” texts, but rarely in the other three texts.

Although the BERT-BiLSTM-CRF model brings higher precision and recall than other models in symptom recognition, it also brings some recognition errors. Qualifiers are misrecognized along with the disease and family history entities, such as “腺囊变不除外 (glandular cyst is not excluded)” and “早发高血压 (early hypertension)”, where the ground truth are “腺囊变 (glandular cyst)” and “高血压 (hypertension)”. Medication entity recognition errors are due to the recognized entities are smaller than actually boundaries, such as “吲达帕胺 (indapamide)” is misrecognized as “达帕胺”. In view of the fact that the BERT model’s misrecognitions are usually due to the qualifiers, the nested entities and using some BERT models trained on clinical and biomedical literature, such as ClinicalBERT and SciBERT, may be considered in our future study to further improve the entity extraction performance.

## Conclusion

In this study, we took the initiative to concern the clinical information extraction for pituitary adenomas based on Chinese EMRs. Entities of symptom, body region, disease, family history, surgery, medication and disease course were determined and recognized from four fine-grained clinical records. To enable machines to intelligently process clinical information, the dictionary-based matching, CRF, BiLSTM-CRF and BERT-BiLSTM-CRF were applied to extract clinical named entities. Experiments demonstrated that the machine learning methods were able to automatically extract clinical entities of pituitary adenomas from EMRs, and the BERT-BiLSTM-CRF model performed the best in both strict and relaxed overall F1 value, reaching 91.27% and 95.57%. Clinical texts noted in EMRs contain abundant diagnosis and treatment information, which is large and unstructured. This study contributes to clinical named entity extraction from Chinese neurosurgical EMRs automatically, which is beneficial to accelerate the secondary applications of clinical unstructured data. The findings could also assist in information extraction in other Chinese medical texts.

## Data Availability

The dataset that support the findings of this study are available from the Institute of Medical information, Chinese Academy of Medical Sciences, but restrictions apply to the availability of these data, which were used under license for the current study, and so are not publicly available. Data are however available from the authors upon reasonable request and with permission of the Peking Union Medical College Hospital.

## Abbreviations

EMR: Electronic Medical Records
CRF: Conditional Random Fields
BiLSTM-CRF: Bidirectional Long Short-Term Memory with CRF
BERT-BiLSTM-CRF: Bidirectional Encoder Representations from Transformers with BiLSTM-CRF
CNN: Convolutional Neural Network
CNLP: Clinical Natural Language Processing
i2b2: Informatics for Integrating Biology & the Bedside
n2c2: National NLP Clinical Challenges
SAR: Shared Annotated Resources
CLEF: Conference and Labs of the Evaluation Forum
CCKS: China Conference on Knowledge Graph and Semantic Computing
CHIP: China Health Information Processing conference
HMM: Hidden Markov Model
SVM: Support Vector Machine
IAA: Inter-Annotator Agreement
JSON: Java Script Object Notation
BIO: Beginning-Inside-Outside
POS: Part of Speech

## Footnotes

1 http://www.openkg.cn/dataset/symptom-in-chinese.

2 This formula determines whether two annotations overlap.
